# Interest in HIV pre-exposure prophylaxis use and associated factors among people who inject drugs in Iran: a nationwide survey in 2023

**DOI:** 10.1038/s41598-026-36329-0

**Published:** 2026-01-25

**Authors:** Hossein Moameri, Soheil Mehmandoost, Fatemeh Tavakoli, Maliheh Sadat Bazrafshani, Naser Nasiri, Hossein Mirzaei, Nasrin Sadidi, Mehrdad Khezri, Ali Akbar Haghdoost, Ali Mirzazadeh, Willi McFarland, Mahkameh Rafiee, Mohammad Karamouzian, Hamid Sharifi

**Affiliations:** 1 Department of Epidemiology, School of Public Health, Shahroud University of Medical Sciences, Shahroud, Iran; 2 HIV/STI Surveillance Research Center, and WHO Collaborating Center for HIV Surveillance, Institute for Futures Studies in Health, Kerman University of Medical Sciences, Kerman, Iran; 3 Department of Public Health, School of Public Health, Jiroft University of Medical Sciences, Jiroft, Iran; 4 Department of Epidemiology, School of Global Public Health, New York University, New York, NY USA; 5 Department of Epidemiology and Biostatistics, University of California, San Francisco, San Francisco, CA USA; 6 Dalla Lana School of Public Health, University of Toronto, Toronto, ON Canada; 7 Institute for Global Health Sciences, University of California, San Francisco, San Francisco, CA USA; 8 Centre on Drug Policy Evaluation, MAP Centre for Urban Health Solutions, St. Michael’s Hospital, ON Toronto, Canada

**Keywords:** Pre-exposure prophylaxis, Harm reduction, People who inject drugs, HIV infection Iran, Diseases, Health care, Medical research, Risk factors

## Abstract

Despite the effectiveness of pre-exposure prophylaxis (PrEP) in reducing HIV incidence, this intervention is inaccessible in Iran. We examined the interest in using PrEP and associated factors among people who inject drugs (PWID) in 2023 using data from 2,174 PWID. The main outcome was interest in using PrEP, which was divided into three categories: interest in using PrEP under any circumstances, interest in using PrEP if provided for free, and no interest in using PrEP. We found that 37.9% of PWID were interested in using PrEP under any circumstances, 48.3% were interested in using PrEP if provided for free, and 13.8% were not interested in using PrEP. Additionally, only 7.7% of participants reported prior awareness of PrEP. Having high school or more education (adjusted relative risk ratios [ARRR]: 1.92; 95% confidence interval [CI]: 1.42, 2.61), having access to opioid agonist treatment (OAT) in the last six months (ARRR: 1.59; 1.13, 2.25), and having sufficient HIV knowledge (ARRR: 2.87; 2.03, 4.06) were positively associated with interest in using PrEP under any circumstances. Similarly, having high school or more education (ARRR: 1.50; 1.10, 2.04), having access to OAT in the last six months (ARRR: 2.63; 1.88, 3.67), and having sufficient HIV knowledge (ARRR: 4.53; 3.23, 6.37) were associated with interest in using PrEP if provided for free. Health insurance was negatively associated with interest in using PrEP under any circumstances (ARRR: 0.64; 0.47, 0.87) and with interest in using PrEP if provided for free (ARRR: 0.33; 0.23, 0.45). The findings show a strong potential for PrEP acceptance, indicating that addressing financial and logistical barriers to free PrEP access could greatly reduce HIV incidence among PWID in Iran.

## Introduction

Pre-exposure prophylaxis (PrEP) has emerged as a significant HIV prevention strategy that has become a central part of national HIV elimination programs in recent years^1,2^. Initially approved by the US Food and Drug Administration in 2012 for HIV-negative adults at high risk of HIV, it has demonstrated 99% effectiveness in preventing HIV transmission when taken consistently^3^. This result led the World Health Organization (WHO) to publish guidelines advocating that PrEPbe included as part of a combined prevention strategy for people at a high risk of contracting HIV^4^. While oral formulations are well-established, PrEP alternatives are continually evolving. The long-acting injectable method may enhance adherence and increase the effectiveness of PrEP^5^. Furthermore, the WHO emphasizes the importance of integrating PrEP as part of a comprehensive approach that encompasses routine HIV testing, counseling, and the promotion of safer sexual practices. This comprehensive approach aims to reduce the stigma linked to HIV prevention strategies and to motivate a more significant number of at-risk individuals to consider PrEP as a suitable preventive method^6^.

The HIV epidemic in Iran is concentrated in key populations, including people who inject drugs (PWID)^7^. According to the latest reports, injection drug use remains one of the main ways HIV is transmitted in Iran^7,8^. Despite the implementation of traditional strategies, such as condom use, reduction of high-risk sexual behaviors, and use of safe needles to prevent HIV transmission, these interventions have not been sufficient to prevent HIV transmission among PWID in Iran ^9^ and in other international contexts^10^. Opioids and stimulants are the primary substances injected by PWID in Iran^11^. Needle and syringe programs (NSPs) and opioid agonist treatment (OAT)  are the primary components of harm reduction programs among PWID in Iran^12,13^. However, studies show that the use of harm reduction programs is still insufficient^14^, and service availability varies significantly by region^13^. Additionally, a recent study estimated that the prevalence of HIV among PWID in Iran is about 3.5%^15^. Biomedical prevention is an additional approach, with one of the most effective and cost-effective recent interventions being PrEP^16–20^.

Although many countries have approved the use of PrEP among key populations, including PWID^21–24^, awareness of and interest in PrEP among this group are still low^25,26^. Furthermore, systematic reviews and meta-analyses focusing on key populations have shown that PWID have the lowest use of PrEP in comparison to other key populations, including men who have sex with men and transgender women^27^. The available evidence indicates that insufficient knowledge and the lack of perceived risk of HIV transmission can create barriers to PrEP uptake among PWID^25,28^. Other factors that can increase interest in using PrEP include access to health services and reducing stigma towards PrEP^26,29^ Limited studies assessed the awareness and interest in using PrEP for HIV prevention among PWID in lower and middle-income countries. Despite growing global adoption, PrEP is not yet used as part of the HIV national prevention strategy in Iran. Before conducting this intervention among PWID, it is important to understand the interest in using PrEP among PWID. The insufficient research conducted within the local context results in a knowledge gap in understanding effective strategies for starting PrEP among PWID. Consequently, this study reports on the interest in using PrEP among PWID in Iran.

## Methods and materials

### Study design and sampling

This analysis utilized data from the fifth national biobehavioral surveillance survey of Iranian PWID, conducted in 14 major cities: Sari (north), Tehran (central north),   Karaj (central north), Tabriz (northwest), Mashhad (northeast), Yazd (central), Kermanshah (west), Khorramabad (west), Dorud (west), Shiraz (south), Ahvaz (southwest), Kerman (southeast), Zahedan (southeast) and Saravan (southeast) across diverse regions. Eligibility criteria included individuals who were 18 years old or older, had reported using at least one injection drug in the previous 12 months, and had a valid referral coupon, except for seeds, following the study’s protocol. Additionally, participants who self-reported as HIV-negative at screening and whose HIV test was negative were recruited for this study. Individuals were recruited using respondent-driven sampling (RDS) between May and August 2023. RDS is a recruitment method that uses long-chain peer referrals to identify and recruit a diverse representation of PWID^30^. The recruitment process began by selecting three seeds per city using a non-random method, with each seed given three referral coupons and trained on how to use them to recruit up to three peers. For all participating cities except Tehran, recruitment was conducted at only one study site. In Tehran, due to the high population, recruitment was conducted across three geographically separate sites, with three seeds initiating the process at each site. The final sample size for each city was determined based on its population proportion, with larger samples allocated to cities with bigger populations. Participants were compensated with 1.5 USD for their participation, followed by three coupons to distribute to their peers for recruitment. An additional 1 USD was provided to participants for each redeemed coupon. This procedure was repeated until the desired sample size was achieved.

### Data collection

Data collection for this study was conducted over four months, from May 2023 to August 2023. All interviews were conducted face-to-face using a standard questionnaire by a gender-matched interviewer in a private room. The questionnaire was in Farsi and included sections on sociodemographic data, history of incarceration, sexual behaviors, HIV status, drug use and injection practices, mental health, and access to harm reduction services, including their interest in using HIV PrEP. After the interviews, participants underwent a brief HIV counseling session and had a whole-blood sample collected via finger-stick by a certified nurse counselor. HIV testing was conducted using the SD-Bioline rapid tests from South Korea; if reactive, the Unigold HIV rapid test was used to confirm the result.

### Study variables

A brief description of PrEP was provided to participants, followed by questions on their interest in using PrEP. This briefing defined PrEP as an HIV prevention strategy, explained that it is available in long-acting injectable, daily oral, and other forms, summarized its effectiveness, and highlighted the importance of continuous adherence to all available formulations. The main outcome of the study included interest in the use of HIV PrEP. Participants were asked about their awareness of PrEP. If they were unfamiliar with it, they were given a brief overview of PrEP before being asked if they would be interested. They were asked a specific question: “Are you interested in the use of HIV PrEP, if it is available?” With response options “interest in using PrEP under any circumstances,” interest in using PrEP if provided for free, and “no interest in using PrEP.” No interest in using PrEP was considered the reference group. Only individuals who self-reported as HIV-negative during screening were asked about their interest in using PrEP.

Covariates of interest included a range of sociodemographic variables, age at interview (< 30 vs. ≥ 30 years old), sex (male vs. female), marital status (currently married vs. single/divorced/widowed), educational level (less than high school vs. high school or more), employment status (unemployed, having a temporary job vs. having a permanent job), having health insurance (yes vs. no), history of homelessness in 12 months (yes vs. no), sex partner (main partners vs. causal partner), lifetime arrest/incarceration (yes vs. no), history of condomless sex with casual partners in last 6 months (yes vs. no), age at first drug use (< 18 vs. ≥18), receptive needle/syringe sharing in last 6 months (yes vs. no), last 6-month daily injection (yes vs. no), last 3-month non-fatal overdose (yes vs. no), last 3-month primary drug injected (opioids vs. stimulants), last 6-month access to OAT (yes vs. no), Lifetime experience of HIV test (yes vs. no), HIV knowledge (insufficient vs. sufficient), and aware of PrEP (yes vs. no). Receptive needle/syringe sharing in the past six months was defined as self-reporting the use of a needle or syringe that had previously been used by another person within the six months prior to the survey. The HIV knowledge was assessed using a standard questionnaire with eight questions^31^. Sufficient knowledge was considered to answer all ten questions correctly.

### Statistical analysis

Descriptive statistics were employed to compare the characteristics of participants stratified by interest in the use of HIV PrEP. The descriptive statistics, including the prevalence estimates shown in Table 1, are based on RDS-weighted data derived with the RDS-II estimator using RDS-A software version 0.42^32^. The RDS-II estimator was used to calculate RDS-weighted point estimates and 95% CI^33^. The number of eligible peers in each participant’s social network was used as the network size parameter for the weighting procedure, and these weights were calculated appropriately. The bivariable and multivariable multinomial logistic regression models were run without RDS-weighted data. First, a bivariable multinomial logistic regression model was used to test the associations between each covariate and the outcome variable. Covariates with P-values of 0.2 or less were included in the multivariable multinomial logistic regression models^34^, and P-values of 0.05 or less were considered statistically significant. Covariates were added to the model one at a time based on their statistical significance and contribution to model fit using a forward stepwise approach for variable selection (entry criterion: *P* < 0.20; retention criterion: P ≤0.05). As a result, only the variables that remained significant in the final model are reported. Additionally, we included covariates that showed a significant association (*P* < 0.2) with any of the non-reference outcome categories in pairwise comparisons against the reference outcome in the final multivariable model. For example, a variable might be linked to “Interest under any circumstances,” but not to “Interest if provided for free.” To assess their fully adjusted effects across all outcome comparisons, these variables were retained in the final model. In the multinomial logistic regression models, no interest in using PrEP was considered the reference group. Crude relative risk ratios (RRR), adjusted relative risk ratios (ARRR), and 95% confidence interval (CI) were reported. Stata 17 was used for all analyses. Under the adjusted covariates, these ARRRs should be interpreted as the relative risk of experiencing one outcome compared to the reference outcome.

**Table 1:** Interest in the use of HIV pre-exposure prophylaxis (PrEP) by sociodemographic characteristics, HIV risk and injection-related factors, and harm reduction utilization among people who inject drugs (PWID) in Iran, 2023.

Variable	Total N (%)	Interest in using PrEP		
		Interest in using PrEP under any circumstances n (RDS^a^ adjusted %)	Interest in using PrEP if provided for free n (RDS adjusted %)	No interest in using PrEPn (RDS adjusted %)
Overall	2,174	824 (37.9)	1,051 (48.3)	299 (13.8)
Current age (years)				
< 30	125 (5.8)	49 (5.6)	54 (5.5)	22 (9.5)
≥ 30	2,049 (94.2)	775 (94.4)	997 (94.5)	277 (90.5)
Sex				
Male	2,084 (95.9)	793 (94.9)	1,012 (95.3)	279 (91.6)
Female	90 (4.1)	31 (5.1)	39 (4.7)	20 (8.4)
Education				
Less than high school	1,441 (66.1)	502 (37.1)	709 (34.5)	223 (21.2)
High school or more	738 (33.9)	321 (62.9)	340 (65.5)	76 (78.8)
Marital status				
Currently married	519 (23.9)	221 (26.2)	192 (22.9)	106 (21.9)
Single/divorced/widowed	1,654 (76.1)	602 (73.8)	859 (77.1)	193 (78.1)
Current employment				
Unemployed	82 (4.7)	20 (1.5)	27 (0.5)	35 (0.8)
Temporary job	1,496 (86.4)	570 (86.1)	732 (93.1)	51 (86.8)
Permanent job	153 (8.9)	72 (12.4)	51 (6.4)	30 (12.4)
Health insurance				
No	1,763 (81.6)	637 (73.8)	923 (89.8)	203 (74.8)
Yes	398 (18.4)	181 (26.2)	124 (10.2)	93 (25.2)
History of homelessness, last year				
No	1,126 (51.9)	487 (64.5)	443 (57.3)	496 (58.6)
Yes	1,046 (48.1)	337 (35.5)	606 (42.7)	103 (41.4)
Sex partner				
Main partner	1,083 (61.5)	405 (69.4)	517 (75.6)	161 (65.5)
Casual partners	679 (38.5)	258 (30.6)	352 (24.4)	69 (34.5)
Lifetime incarceration				
No	637 (29.3)	288 (38.8)	267 (42.5)	82 (38.0)
Yes	1,535 (70.7)	535 (61.2)	783 (57.5)	217 (62.0)
History of condomless sex with casual partners in last 6 months				
No	509 (71.3)	226 (79.9)	246 (61.0)	37 (65.6)
Yes	204 (28.7)	48 (20.1)	119 (39.0)	37 (34.4)
Age at first drug use, years				
< 18	1,462 (67.2)	566 (72.9)	695 (67.3)	201 (61.6)
≥18	712 (32.8)	285 (27.1)	356 (32.7)	98 (38.4)
Receptive needle/syringe sharing, last 6 months				
No	1,849 (86.8)	657 (91.1)	932 (94.9)	260 (87.9)
Yes	279 (13.2)	151 (8.9)	103 (5.1)	25 (12.1)
Daily injection in last 6 months				
No	1,099 (51.7)	459 (66.4)	463 (58.5)	177 (70.9)
Yes	1,025 (48.3)	357 (33.6)	551 (41.5)	177 (29.1)
Experience of non-fatal overdose, last year				
No	2,010 (93.5)	752 (91.3)	991 (95.3)	267 (94.9)
Yes	140 (6.5)	64 (8.7)	50 (4.7)	26 (5.01)
Primary drug injected, last 3 months				
Stimulants	68 (3.9)	29 (7.9)	19 (3.8)	20 (15.6)
Opioids	1,640 (96.1)	611 (92.1)	821 (96.2)	208 (84.4)
Opioid agonist treatment, last 6 months				
No	637 (29.3)	205 (21.6)	379 (27.4)	53 (22.4)
Yes	1,537 (70.7)	619 (78.4)	672 (72.6)	246 (77.6)
HIV knowledge^b^				
Insufficient	1,311 (60.3)	520 (65.1)	539 (66.7)	252 (77.3)
Sufficient	863 (39.7)	304 (34.9)	512 (33.3)	47 (22.7)
History of HIV test, lifetime				
No	412 (19.0)	153 (25.8)	154 (22.2)	105 (32.1)
Yes	1,762 (81.0)	671 (74.2)	897 (77.8)	194 (67.9)
Aware of PrEP				
No	1,987 (92.3)	732 (90.4)	990 (93.2)	265 (89.5)
Yes	164 (7.7)	82 (9.6)	57 (6.79)	25 (10.5)

^a^Respondent-driven sampling.

^b^Measured using an 8-item set of questions covering basic knowledge of HIV/AIDS transmission and prevention.

### Ethical considerations

Study staff ensured confidentiality by using anonymous questionnaires and obtaining informed consent from participants for data collection. They were told that their decision to decline participation would not affect them in any way. They were assured that they could refuse to answer any questions they wanted and stop the interview at any time. The Kerman University of Medical Sciences research ethics committee reviewed and approved the protocol and procedures for the current study (Ethics Code: IR.KMU.REC.1401.216). In addition, all methods were performed in accordance with the relevant guidelines and regulations.

## Results

### Characteristics of the sample

Among 2,174 PWID, most participants (95.9%) were men and aged more than 30 years old (94.2%) (Table 1). About two-thirds (66.1%) had less than a high school education, and 76.1% were single, divorced, or widowed. Most participants (86.4%) had temporary employment and did not have health insurance (81.6%). Over two-thirds (70.7%) had been incarcerated in their lifetime, and 48.1% had a history of homelessness in the last year. Nearly half (48.3%) reported daily injections in the last six months, with opioids as the primary drug injected in the last three months (96.1%). Only 7.7% were aware of PrEP.

### Interest in using PrEP

The prevalence of interest in using PrEP under any circumstances, interest in using PrEP if provided for free, and no interest in using PrEP use was 37.9% (95% CI: 35.8, 39.9), 48.3% (95% CI: 46.2, 50.4), and 13.8% (95% CI: 12.3, 15.2), respectively (Table 1).

#### Factors associated with interest in using PrEP under any circumstances

Bivariable multinominal logistic regression showed that interest in using PrEP under any circumstances was significantly associated with being male, being single/divorced/widowed, having a high school education or more, having a temporary or permanent job, not having health insurance, having a casual partner, not having lifetime incarceration, not having history of condomless sex with casual partners in last six months, having needle/syringe sharing in the previous six months, access to OAT in the last six months, having a history of HIV test, and having sufficient HIV knowledge (Table 2).

**Table 2:** Bivariable multinominal logistic regression of associated factors with interest in the use of HIV preexposure prophylaxis (PrEP) and associated factors among people who inject drugs in Iran, 2023, (*n* = 2,174).

Variable	Interest in using PrEP under any circumstances		Interest in using PrEP if provided for free	
	Crude risk ratios^a^ (95% CI^b^)	P- value	Crude risk ratios^a^ (95% CI^b^)	P- value
Current age (years)				
< 30	Ref		Ref	
≥ 30	1.25 (0.75–2.11)	0.391	1.46 (0.87–2.11)	0.144
Sex				
Male	Ref		Ref	
Female	0.54 (0.30–0.97)	0.040	0.53 (0.30–0.93)	< 0.001
Marital status				
Currently married	Ref		Ref	
Single/divorced/widowed	1.49 (1.12–1.98)	0.005I	2.45 (1.84–3.26)	< 0.001
Education				
Less than high school	Ref		Ref	
High school or more	1.87 (1.39–2.54)	< 0.001	1.40 (1.05–1.88)	0.021
Current employment				
Unemployed	Ref		Ref	
Temporary job	5.14 (2.89–9.11)	< 0.001	4.89 (2.88–8.27)	< 0.001
Permanent job	4.20 (2.09–8.41)	< 0.001	2.20 (1.12–4.32)	0.022
Health insurance				
No	Ref		Ref	
Yes	0.62 (0.46–0.83)	< 0.002	0.29 (0.21–0.39)	< 0.001
History of ever homelessness in the last year				
No	Ref		Ref	
Yes	1.31 (1.00- 2.09)	< 0.050	2.10 (1.77–2.49)	< 0.001
Sex partner				
Main partners	Ref		Ref	
Casual partner	1.48 (1.07–2.05)	0.016	1.58 (1.16–2.17)	0.004
Lifetime arrest/incarceration				
No	Ref		Ref	
Yes	0.67 (0.52–0.93)	0.017	1.10 (0.82–1.48)	0.487
History of condomless sex with casual partners in last 6 months				
No	Ref		Ref	
Yes	0.21 (0.12–0.36)	< 0.001	0.28 (0.17–0.80)	< 0.005
Age at first drug use				
< 18	Ref		Ref	
≥ 18	0.93 (0.70–1.24)	0.641	1.05 (0.79–1.38)	0.723
Receptive needle/syringe sharing, last 6 months				
No	Ref		Ref	
Yes	2.39 (1.52–3.73)	< 0.001	1. 41 (0.72–1.81)	0.551
Daily injection in the last 6 months				
No	Ref		Ref	
Yes	1.17 (0.89–1.54)	0.240	1.80 (1.38–2.34)	< 0.001
Experience of non-fatal overdose, last 3 months				
Yes	Ref		Ref	
No	0.87 (0.54–1.40)	0.580	0.51 (0.31–0.84)	0.009
Primary drug injected, last 3 months				
Stimulants	Ref		Ref	
Opioids	1.23 (0.91–1.44)	0.070	1.21 (0.99–1.46)	0.062
Access to opioid agonist therapy in the last 6 months				
No	Ref		Ref	
Yes	1.53 (1.09–2.15)	0.012	2.61 (1.89–3.61)	< 0.001
HIV knowledge				
Insufficient	Ref		Ref	
Sufficient	3.13 (2.22–4.41)	< 0.001	5.09 (3.64–7.11)	< 0.001
History of HIV test, lifetime				
No	Ref		Ref	
Yes	2.37 (1.79–3.18)	< 0.001	3.15 (2.35–4.22)	< 0.001
Aware of PrEP				
No	Ref		Ref	
Yes	1.18 (0.74–1.89)	0.473	0.61 (0.37–1.01)	0.051

^a^The reference group for the risk ratios was not interested in using PrEP.

^b^Confidence intervals.

The multinomial logistic regression showed that interest in using PrEP under any circumstances was significantly associated with high school education (ARRR: 1.92; 95% CI: 1.42, 2.61), access to OAT in the last six months (ARRR: 1.59; 95% CI: 1.13, 2.25), and sufficient HIV knowledge (ARRR: 2.87; 95% CI: 2.03, 4.06). However, health insurance was negatively associated with interest in using PrEP under any circumstances (ARRR: 0.64; 0.47, 0.87) (Table 3).

**Table 3:** Multivariable nominal logistic regression of associated factors with interest in the use of HIV preexposure prophylaxis (PrEP) and associated factors among people who inject drugs in Iran, 2023, (*n* = 2,174).

Variable	Interest in using PrEP under any circumstances		Interest in using PrEP if provided for free	
	Adjusted risk ratios ^a^ (95% CI^b^)	P-value	Adjusted risk ratios ^a^ (95% CI^b^)	P-value
Education				
Less than high school	Ref		Ref	
High school or more	1.92 (1.42–2.61)	< 0.001	1.50 (1.10–2.04)	0.010
Health insurance				
No	Ref		Ref	
Yes	0.64 (0.47–0.87)	0.004	0.33 (0.23–0.45)	< 0.001
Access to opioid agonist treatment in the last 6 months				
No	Ref		Ref	
Yes	1.59 (1.13–2.25)	0.008	2.63 (1.88–3.67)	< 0.001
HIV knowledge				
Insufficient	Ref		Ref	
Sufficient	2.87 (2.03–4.06)	< 0.001	4.53 (3.23–6.37)	< 0.001

^a^The reference group for the risk ratios was not interested in using PrEP.

^b^Confidence intervals.

#### Factors associated with interest in using PrEP if provided for free

Bivariable multinominal logistic regression showed that interest in using PrEP, if provided for free, was significantly associated with being male, being single/divorced/widowed, having a high school education and more, having a temporary or permanent job, not having health insurance, having a history of homelessness in the last year, having a casual partner, not having a history of condomless sex with casual partners in last six months, having a daily injection in the last six months, access to OAT in the last six months, having experience of non-fatal overdose in last three months, primary drug injected in the past 3 months, having a history of HIV test, having sufficient HIV knowledge and aware of PrEP (Table 2).

The multinomial logistic regression showed that interest in using PrEP if provided for free was significantly associated with high school education (ARRR: 1.50; 95% CI: 1.10, 2.04),   having access to OAT in the last six months (ARRR: 2.63; 95% CI: 1.88, 3.67), and having sufficient HIV knowledge (ARRR: 4.53; 95% CI: 3.23, 6.37). Health insurance was negatively associated with interest in using PrEP if provided for free (ARRR: 0.33; 0.23, 0.45) (Table 3).

## Discussion

We found that only one in 13 PWID in Iran were previously aware of PrEP. Once the intervention was explained to them, nearly 40% were interested in using PrEP under any circumstances, and nearly half were interested in using PrEP if provided for free. We also found that interest in the use of HIV PrEP regardless of cost was significantly associated with having a high school education or higher, not having health insurance, having access to OAT in the last six months, and having sufficient HIV knowledge. When the same factors were examined for interest contingent on free provision, the associations persisted;  high school education or higher, lack of health insurance, OAT access in the last six months, and sufficient HIV knowledge all remained significantly associated with interest in PrEP use if provided at no cost.

The fact that approximately 40% of respondents expressed interest in using PrEP regardless of having to pay for it underscores the strength of the perceived benefits in reducing the risk of HIV transmission. Therefore, the interest in using PrEP is a significant finding for policymakers. Although recent studies showed notable interest in using PrEP, especially if cost barriers were removed^35,36^, the interest in using PrEP in our study was lower than that observed in other studies, with interest rates of 59% in San Francisco^26^, 63% in Baltimore^35^, and 65% in Connecticut^37^. Low baseline awareness cannot be the only explanation for this difference because our modeling showed that basic awareness alone was not significantly associated with interest. Instead, this suggests that a deeper level of precise, comprehensive knowledge may be required to transition from basic awareness to genuine interest in using PrEP, which may not have been achieved in our study (where familiarity was below 8%). Evidence that stigma continues to be a barrier to interest in using PrEP further complicates this^38^. This finding is consistent with previous research in Iran, demonstrating that stigma is one of the main barriers to PrEP uptake among high-risk groups for HIV, such as PWID^39^. Furthermore, studies have demonstrated that factors such as PrEP awareness, knowledge, perceived HIV risk, perceived need for PrEP, and social factors play crucial roles in individuals’ intention to use PrEP^40,41^. A previous national survey showed that harm reduction programs, such as HIV testing, are still inadequate, which may reflect gaps in perceived HIV risk or awareness among PWID^42^. These findings underscore the importance of addressing social, financial, and informational barriers to enhance the uptake of PrEP and reduce the incidence of HIV among key populations, particularly PWID. Additionally, our findings suggest that removing financial barriers could immediately produce a substantial impact at the population level that surpasses what awareness campaigns alone can achieve. Moreover, it is essential to address sociocultural barriers to PrEP utilization, as neglecting this issue could reduce the potential advantages of biomedical prevention strategies.

Our multivariable multinomial logistic regression analysis highlighted the significant effect of education level and HIV knowledge on individuals’ interest in using PrEP as a preventative measure for HIV transmission. The results indicated that individuals with higher levels of education were more inclined to consider using PrEP than those with lower education. As shown in previous studies, these results show a potential link between education and health literacy in influencing preventive health behavior^43,44^. Moreover, the positive relationship between HIV knowledge and interest in using PrEP, highlights the critical role of education in shaping individuals’ attitudes and behaviors toward preventive strategies^25,45^. Education may foster an understanding of complex health information, which facilitates comprehension of how PrEP may be used to prevent HIV, which in turn may drive interest. By implementing targeted educational interventions, we can improve individuals’ awareness of HIV and PrEP, thereby increasing their interest in using PrEP and contributing to improved public health outcomes.

In addition, the multinomial logistic regression showed that interest in using PrEP was significantly associated with having access to OAT. Furthermore, the association between OAT access and increased interest in using PrEP underscores the potential for integrating HIV prevention efforts. Some studies showed that individuals with a history of OAT utilization might benefit from targeted interventions integrating PrEP education within the existing healthcare services^37,46^. This finding is especially important in the Iranian context, where OAT serves as a primary harm reduction delivery channel for PWID and is mainly administered by government-supported treatment centers^47^. This association highlights the effectiveness of integrated programs in preventing HIV. Since OAT users already receive structured healthcare services, these facilities represent optimal settings to include PrEP education and improve access in Iran^48^. Additionally, individuals undergoing OAT often engage with healthcare services focused on substance use treatment and preventive health initiatives. Such involvement creates an environment where conversations about HIV prevention, including PrEP, are more likely to occur. Moreover, those in OAT programs may be more aware of their health risks and the significance of preventive measures, which can boost their interest in obtaining PrEP. Furthermore, people who are on OAT are also probably accustomed to administering their medications on a regular schedule for a chronic condition^49^. This prior experience may reduce the perceived barriers associated with treatment frequency and adherence challenges frequently connected to long-term preventive measures such as PrEP, thereby enhancing their willingness to use it. Integrating PrEP education within OAT services may enhance knowledge of PrEP and facilitate access to this method. By implementing this approach, we can leverage the existing framework of OAT services to enhance interest and access to PrEP, thereby strengthening overall HIV prevention efforts.

We also found that individuals with health insurance were significantly less likely to express interest in using PrEP, whether provided for free or at a cost. This finding highlights the role of health insurance coverage in shaping perceptions of preventive healthcare services such as PrEP. The inverse relationship between health insurance status and interest in PrEP uptake underscores the need for further investigation into the underlying factors driving this disparity. In contrast to the results of our study, previous studies emphasized the positive role of having health insurance in key populations^36,50^ receiving PrEP. This difference suggests that health insurance among PWID in Iran functions primarily as a proxy marker for higher socioeconomic status (SES) and established engagement with formal healthcare systems, rather than merely indicating affordability or access barriers as is often the case in other contexts. Individuals with formal employment usually have comprehensive coverage through the Social Security Organization in Iran. However, marginalized populations often depend on subsidized national health insurance plans that might offer more limited coverage. Considering this SES indicator, there are several reasons why insured individuals may be less interested in using PrEP: they may have a lower personal risk profile compared to uninsured groups, or they could have a higher baseline level of general health literacy, decreasing their perceived need for a new intervention like PrEP. Addressing these barriers through focused interventions and education campaigns could help reduce the PrEP uptake gap among those with health insurance, resulting in more equitable access to HIV prevention programs across all populations. Furthermore, collaborating with health insurance providers to deliver PrEP-related educational materials to their members can enhance awareness and understanding of this preventive method.

Our study has several limitations. First, the cross-sectional design of this study precludes the establishment of causal relationships. Second, a noticeable proportion of participants surveyed were introduced to PrEP for the first time through this study, which may influence their perceptions of interest in using PrEP. Third, our findings are prone to recall and social desirability biases. Lastly, we could not evaluate the perceived risk of HIV acquisition during the data collection process. Because of this limitation, our model cannot show how this factor impacts preventive behaviors (e.g., using PrEP). To better understand the barriers and motivators of PrEP use, future studies should include validated tools to assess risk perception in this group.

## Conclusions

While prior awareness of PrEP was low, most PWID were interested in using PrEP once made aware of its potential efficacy in preventing HIV acquisition via sexual contact or shared injection equipment. This finding demonstrates to policymakers the importance of integrating PrEP into national harm reduction programs as a means to reduce HIV incidence in this key population by lowering costs and expanding access. Our country, therefore, must examine the conditions for the inclusion of PrEP in the national harm reduction program. A complex association between education level, access to OAT, HIV knowledge, and health insurance coverage affects people’s motivation to use PrEP as a preventive intervention for HIV transmission. These findings emphasize the need to overcome educational, informational, and access barriers to increase PrEP use and support effective HIV prevention strategies among key populations. This can be achieved through educational campaigns and collaboration with various organizations.

## Data Availability

All data generated or analyzed during this study are included in this published article.

## Abbreviations

PrEP: Pre-exposure prophylaxis
WHO: World Health Organization
PWID: People who inject drugs
RDS: Respondent-driven sampling
RRR: Crude relative risk ratios
ARRR: Adjusted relative risk ratios
OAT: Opioid agonist treatment

## References

[1] Roberts, D. A., Bridenbecker, D., Haberer, J. E., Barnabas, R. V. & Akullian, A. The impact of prevention-effective PrEP use on HIV incidence: a mathematical modelling study. *J. Int. AIDS Soc.* **25**(11), e26034 (2022). 36385504 10.1002/jia2.26034 PMC9670193

[2] Tassi, M-F. et al. PrEP monitoring and HIV incidence after PrEP initiation in france: 2016–18 nationwide cohort study. *J. Antimicrob. Chemother.* **76**(11), 3002–3008 (2021). 34293116 10.1093/jac/dkab263

[3] Zhao, A. et al. Pharmacy-based interventions to increase use of HIV pre-exposure prophylaxis in the united states: a scoping review. *AIDS Behav.* 1–16 (2022). 10.1007/s10461-021-03494-4 PMC8527816 34669062

[4] WHO. *Consolidated Guidelines on the Use of Antiretroviral Drugs for Treating and Preventing HIV Infection: Recommendations for a Public Health Approach* (World Health Organization, 2016). 27466667

[5] Algarin, A. B. & Shrader, C. H. Advancing PrEP for HIV prevention: innovations and the imperative to preserve comprehensive care. *BMC Glob Public. Health*. **3**(1), 9 (2025). 39885612 10.1186/s44263-025-00125-1 PMC11784111

[6] WHO. WHO implementation tool for pre-exposure prophylaxis of HIV infection: provider module for oral and long-acting PrEP 2024 [Available from: https://www.who.int/publications/i/item/9789240097230

[7] SeyedAlinaghi, S. et al. HIV in iran: onset, responses, and future directions. *Aids* **35**(4), 529–542 (2021). 33252485 10.1097/QAD.0000000000002757 PMC7924262

[8] Fahimfar, N., Sedaghat, A., Hatami, H., Kamali, K. & Gooya, M. Counseling and harm reduction centers for vulnerable women to HIV/AIDS in Iran. *Iran. J. Public. Health*. **42**(Supple1), 98 (2013). 23865025 PMC3712587

[9] Omidi, T., Oshnouei, S., Mahdi-Akhgar, M., Mohammadian-Khoshnoud, M. & Mohammadi, Y. Barriers to condom use among female sex workers: aA systematic review and meta-analysis. *Curr. Womens Health Rev.* **19**(3), 93–107 (2023).

[10] Godfrey-Faussett, P., Frescura, L., Abdool Karim, Q., Clayton, M. & Ghys, P. D. HIV prevention for the next decade: Appropriate, person-centred, prioritised, effective, combination prevention. *PLoS Med.* **19**(9), e1004102 (2022). 36156593 10.1371/journal.pmed.1004102 PMC9550175

[11] Abdolahinia, Z. et al. Correlates of duration between initial drug use and first drug injection among people who inject drugs in Iran, 2020. *BMC Public. Health*. **25**(1), 1229 (2025). 40170019 10.1186/s12889-025-22357-4 PMC11959802

[12] Ekhtiari, H. et al. The evolution of addiction treatment and harm reduction programs in iran: a chaotic response or a synergistic diversity? *Addiction* **115**(7), 1395–1403 (2020). 31737965 10.1111/add.14905

[13] Rahnama, R. et al. Access to harm reduction programs among persons who inject drugs: findings from a respondent-driven sampling survey in Tehran, Iran. *Int. J. Drug Policy*. **25**(4), 717–723 (2014). 24974367 10.1016/j.drugpo.2014.05.013 PMC4231781

[14] Tavakoli, F. et al. Double counting of clients using services in iran: implications for assessing the reach of harm reduction programs. *Harm Reduct. J.* **20**(1), 111 (2023). 37587473 10.1186/s12954-023-00851-5 PMC10429072

[15] Khezri, M. et al. HIV prevalence and related behaviors among people who inject drugs in Iran from 2010 to 2020. *AIDS Behav.* **26**(9), 2831–2843 (2022). 35195820 10.1007/s10461-022-03627-3

[16] Choi, H. et al. Cost-effectiveness analysis of pre-exposure prophylaxis for the prevention of HIV in men who have sex with men in South korea: a mathematical modelling study. *Sci. Rep.* **10**(1), 14609 (2020). 32884082 10.1038/s41598-020-71565-y PMC7471951

[17] Chou, R. et al. Preexposure prophylaxis for the prevention of HIV infection: evidence report and systematic review for the US preventive services task force. *Jama* **321**(22), 2214–2230 (2019). 31184746 10.1001/jama.2019.2591

[18] Marcus, J. L. et al. Preexposure prophylaxis for HIV prevention in a large integrated health care system: adherence, renal safety, and discontinuation. *J. Acquir. Immune Defic. Syndr.* **73**(5), 540–546 (2016). 27851714 10.1097/QAI.0000000000001129 PMC5424697

[19] Moameri, H. et al. Cost-effectiveness of HIV pre-exposure prophylaxis among female sex workers in Iran. *Sci. Rep.* **15**(1), 7747 (2025). 40044976 10.1038/s41598-025-92099-1 PMC11882906

[20] Guy, D. et al. The HIV pre-exposure prophylaxis continuum of care among women who inject drugs: A systematic review. *Front. Psichiatry*. **13**, 951682 (2022). 10.3389/fpsyt.2022.951682 PMC9459118 36090369

[21] Fauci, A. S., Redfield, R. R., Sigounas, G., Weahkee, M. D. & Giroir, B. P. Ending the HIV epidemic: A plan for the united States. *JAMA* **321**(9), 844–845 (2019). 30730529 10.1001/jama.2019.1343

[22] Galea, J. T., Baruch, R. & Brown, B. Inverted exclamation markprep Ya! Latin America wants PrEP, and Brazil leads the way. *Lancet HIV*. **5**(3), e110–e2 (2018). 29467099 10.1016/S2352-3018(18)30011-0

[23] Annequin, M. et al. Are PrEP services in France reaching all those exposed to HIV who want to take PrEP? MSM respondents who are eligible but not using PrEP (EMIS 2017). *Aids Care*. **32**(sup2), 47–56 (2020). 32189518 10.1080/09540121.2020.1739219

[24] Muhumuza, R. et al. Exploring perceived barriers and facilitators of PrEP uptake among young people in Uganda, Zimbabwe, and South Africa. *Arch. Sex. Behav.* **50**(4), 1729–1742 (2021). 33954824 10.1007/s10508-020-01880-y PMC8213546

[25] Bazzi, A. R. et al. Limited knowledge and mixed interest in pre-exposure prophylaxis for HIV prevention among people who inject drugs. *AIDS Patient STD*. **32**(12), 529–537 (2018). 10.1089/apc.2018.0126 PMC6300044 30311777

[26] Walters, S. M., Kral, A. H., Simpson, K. A., Wenger, L. & Bluthenthal, R. N. HIV pre-exposure prophylaxis prevention awareness, willingness, and perceived barriers among people who inject drugs in Los Angeles and San Francisco. *Subst. Use Misuse*. **55**(14), 2016–2018 (2020). 10.1080/10826084.2020.1823419 PMC7665852 32962490

[27] Kamitani, E. et al. Growth in proportion and disparities of HIV PrEP use among key populations identified in the united States National goals: systematic review and meta-analysis of published surveys. *J. Acquir. Immune Defic. Syndr.* **84**(4), 379–386 (2020). 32205721 10.1097/QAI.0000000000002345 PMC8022293

[28] Biello, K. et al. Perspectives on HIV pre-exposure prophylaxis (PrEP) utilization and related intervention needs among people who inject drugs. *Harm Reduct. J.* **15**, 1–12 (2018). 30419926 10.1186/s12954-018-0263-5 PMC6233595

[29] Tavakoli, F. et al. HIV-related stigma among healthcare providers in different healthcare settings: a cross-sectional study in kerman, Iran. *Int. J. Health Policy Manag*. **9**(4), 163–169 (2020). 32331496 10.15171/ijhpm.2019.92 PMC7182146

[30] Solomon, S. S. et al. Respondent-driven sampling for identification of HIV-and HCV-infected people who inject drugs and men who have sex with men in india: a cross-sectional, community-based analysis. *PLoS Med.* **14**(11), e1002460 (2017). 29182638 10.1371/journal.pmed.1002460 PMC5705124

[31] UNAIDS. Monitoring the Declaration of Commitment on HIV/AIDS: Guidelines on construction of core indicators – 2008 reporting. Geneva, Switzerland 2077 [Available from: http://www.unaids.org/sites/default/files/mediaasset/jc1318

[32] Handcock, M., Fellows, I. & Gile, K. Hard-to-Reach Population Methods Research Group (2014).

[33] Volz, E. & Heckathorn, D. D. Probability based Estimation theory for respondent driven sampling. *J. Off Stat.* **24**(1), 79 (2008).

[34] Maldonado, G. & Greenland, S. Simulation study of confounder-selection strategies. *Am. J. Epidemiol.* **138**(11), 923–936 (1993). 8256780 10.1093/oxfordjournals.aje.a116813

[35] Sherman, S. G. et al. PrEP awareness, eligibility, and interest among people who inject drugs in Baltimore, Maryland. *Drug Alcohol Depend.* **195**, 148–155 (2019). 30639794 10.1016/j.drugalcdep.2018.08.014 PMC6436943

[36] Sun, Z. et al. Increasing awareness of HIV pre-exposure prophylaxis (PrEP) and willingness to use HIV PrEP among men who have sex with men: a systematic review and meta‐analysis of global data. *J. Int. AIDS Soc.* **25**(3), e25883 (2022). 35255193 10.1002/jia2.25883 PMC8901150

[37] Ni, Z. et al. Willingness to initiate pre-exposure prophylaxis (PrEP) and its use among opioid-dependent individuals in drug treatment. *Drug Alcohol Depend.* **219**, 108477 (2021). 33422864 10.1016/j.drugalcdep.2020.108477 PMC7946167

[38] Eaton, L. A. et al. Stigma and conspiracy beliefs related to pre-exposure prophylaxis (PrEP) and interest in using PrEP among black and white men and transgender women who have sex with men. *AIDS Behav.* **21**, 1236–1246 (2017). 28108878 10.1007/s10461-017-1690-0 PMC5926187

[39] Moameri, H. et al. Facilitators and barriers of HIV pre-exposure prophylaxis use among four key populations in Iran. *BMC Health Serv. Res.* **24**(1), 1433 (2024). 39563358 10.1186/s12913-024-11933-w PMC11575091

[40] Ayangeakaa, S. D. et al. Understanding influences on intention to use pre-exposure prophylaxis (PrEP) among African American young adults. *J. Racial Ethn. Disparities*. **10**(2), 899–910 (2023). 10.1007/s40615-022-01278-7 35290648

[41] Thomann, M., Grosso, A., Zapata, R. & Chiasson, M. A. WTF is PrEP?’: attitudes towards pre-exposure prophylaxis among men who have sex with men and transgender women in new York City. *Cult. Health Sex.* **20**(7), 772–786 (2018). 28982311 10.1080/13691058.2017.1380230 PMC6217837

[42] Roshanfekr, P. et al. Life-time HIV testing among people who inject drugs in iran: results from the National rapid assessment and response survey. *Front. Public. Health*. **11**, 1253407 (2023). 37915820 10.3389/fpubh.2023.1253407 PMC10616789

[43] Cunha GHd, Galvão, M. T. G., Pinheiro, P. N. C. & Vieira, N. F. C. Health literacy for people living with HIV/Aids: an integrative review. *Rev. Bras. Enferm*. **70**, 180–188 (2017). 28226058 10.1590/0034-7167-2015-0052

[44] Slurink, I. A., Götz, H. M., van Aar, F. & van Benthem, B. H. Educational level and risk of sexually transmitted infections among clients of Dutch sexual health centres. *Int. J. STD AIDS*. **32**(11), 1004–1013 (2021). 33993803 10.1177/09564624211013670

[45] Shamu, S. et al. Pre-exposure prophylaxis (PrEP) awareness, attitudes and uptake willingness among young people: gender differences and associated factors in two South African districts. *Glob Health Action*. **14**(1), 1886455 (2021). 33606603 10.1080/16549716.2021.1886455 PMC7899653

[46] Page, K. et al. Biomedical HIV prevention including pre-exposure prophylaxis and opiate agonist therapy for women who inject drugs: state of research and future directions. *J. Acquir. Immune Defic. Syndr.* **69**, S169–S75 (2015). 25978484 10.1097/QAI.0000000000000641 PMC4491435

[47] Noroozi, A. et al. Factors influencing engagement and utilisation of opium tincture-assisted treatment for opioid use disorder: A qualitative study in Tehran, Iran. *Drug Alcohol Rev.* **41**(2), 419–429 (2022). 34309108 10.1111/dar.13357

[48] Moameri, H. et al. Barriers and solutions to implement HIV pre-exposure prophylaxis program among key populations in iran: a qualitative study using causal layered analysis. *Health Sci. Rep.* **8**(7), e70968 (2025). 40626192 10.1002/hsr2.70968 PMC12230372

[49] Tetrault, J. M. & Fiellin, D. A. Current and potential Pharmacological treatment options for maintenance therapy in opioid-dependent individuals. *Drugs* **72**(2), 217–228 (2012). 22235870 10.2165/11597520-000000000-00000 PMC3701303

[50] Ogunbajo, A., Iwuagwu, S., Williams, R., Biello, K. & Mimiaga, M. J. Awareness, willingness to use, and history of HIV PrEP use among gay, bisexual, and other men who have sex with men in Nigeria. *PloS One*. **14**(12), e0226384 (2019). 31851722 10.1371/journal.pone.0226384 PMC6919589

